# A Multi-sourced Data Analytics Approach to Measuring and Assessing Biopsychosocial Complexity: The Vancouver Community Analytics Tool Complexity Module (VCAT-CM)

**DOI:** 10.1007/s10597-019-00417-5

**Published:** 2019-06-08

**Authors:** Ali Rafik Shukor, Ronald Joe, Gabriela Sincraian, Niek Klazinga, Dionne Sofia Kringos

**Affiliations:** 1 Department of Public Health, Amsterdam Public Health Research Institute, University of Amsterdam, Amsterdam UMC, Meibergdreef 9, 1105 AZ Amsterdam, The Netherlands; 2 Vancouver Coastal Health (VCH), 520 West 6th Ave, Vancouver, BC Canada

**Keywords:** Biopsychosocial, Complexity, Data analytics, Primary Health Care, Community Health Center, Vancouver Coastal Health

## Abstract

Operationalization of the fundamental building blocks of primary care (i.e. empanelment, team-based care and population management) within the context of Community Health Centers requires accurate and real-time measures of biopsychosocial complexity, at both client and population-levels. This article describes the conceptualization, design and development of a novel software tool (the *VCAT*-*Complexity Module*) that can calculate and report real-time person-oriented biopsychosocial complexity profiles, using multiple data sources. The tool aligns with a profile approach to conceptualizing health outcomes, and represents a potentially significant advance over disease-oriented complexity assessment tools. The results and face validity of the software’s complexity score outputs are discussed, along with their practical implications on functions related to the development of primary care within Vancouver Coastal Health, a Canadian Regional Health Authority.

## Introduction

### Context

Vancouver Coastal Health (VCH) is one of six publicly funded Regional Health Authorities in British Columbia (BC, Canada). Its public Community Health Centers (CHCs) are officially mandated to provide primary care services to its jurisdiction’s clients with complex biopsychosocial needs—particularly those not rostered to, or without regular access (i.e. “unattached”) to a primary care clinic or provider (Shukor et al. 2018).

The biopsychosocial complexity profile of VCH’s CHC clients—and more fundamentally, the inability to accurately and comprehensively model it in an efficient and timely manner—present serious challenges to meeting the mandate (Shukor et al. 2018). The complexity, epidemiological and health care utilization profiles of VCH’s socioeconomically marginalized population are not appropriately or adequately represented by existing databases (e.g. hospital, community [fee for service] primary care, home care, pharmaceutical and diagnostic databases) or population and health care utilization profiles (Local Health Area Profiles 2016; Primary and Community Care Profile: Your Community (Vancouver Downtown Eastside) 2017). This is due to factors related to significant health care access barriers facing transient and marginalized populations, intrinsic limitations of disease-oriented medical record classification standards (e.g. the International Classification of Disease), inaccuracies associated with professional judgment, and poor standards of record keeping within health and social services sectors (Somers et al. 2015, 2016; Rosendal et al. 2015; Soler and Okkes 2012). Existing databases are often siloed, with significant data quality, completeness, accessibility, and analytics and reporting issues. This is not just an issue in BC, but has been shown to be problematic in other countries as well (Green et al. 2015; Singer et al. 2017; Van der Bij et al. 2017; Birtwhistle and Williamson 2015).

Existing organizational reports and academic studies of the region’s health and social services depict marginalized, multi-ethnic and transient populations with high incidence and prevalence rates of mental illness, substance use, trauma, and communicable and non-communicable illness and disease (Somers et al. 2015, 2016; Parpouchi et al. 2017; Carnegie Community Action Project 2018; Linden et al. 2013). Many clients (including a sizeable minority of frail seniors) are low-income, food-insecure, housing-insecure or homeless and face difficulties associated with access to social and health care services (Carnegie Community Action Project 2018; BC Non-Profit Housing Association & M.Thomson Consulting 2017). Clients present with histories of challenging patient–provider relationships (e.g., the “difficult” patient that has been recently “fired” by their GP), and are often described as over-serviced but under-served, while some are both underserved and underserviced (Shukor et al. 2018).

The severe health and social impact of high biopsychosocial complexity is manifested through statistics of health outcomes and service utilization. For example, between 2006 and 2013, the median age of death for a homeless person in BC was found to be between 40 and 49, which is approximately half of the provincial average life expectancy. Accidental deaths, suicide and homicide accounted for 47.7, 12.5 and 3.9% of all homeless deaths in BC, compared to 18.3, 6.3 and 1.5% of general population deaths, respectively (Condon and McDermid 2014). A study of high frequency health and social service users from Vancouver’s Downtown Eastside neighborhood with community and custody sentences found that 323 clients incurred a cost of $26.5 million to public health and social services, over a period of 5 years (Somers et al. 2015). 99% had been diagnosed with at least one mental disorder, and 82% had co-occurring substance use and mental disorders (Somers et al. 2015).

### Measuring and Assessing Biopsychosocial Complexity: The Questionnaire Approach

The complexity profiles of clients pose significant challenges relating to operationalizing the fundamental “*building blocks of high performing primary care*” framework, particularly within the context of CHCs (Shukor et al. 2018; Bodenheimer et al. 2014; Quinn et al. 2013; Anderson and Olayiwola 2012; Reibling and Rosenthal 2016). The framework, developed by the University of California’s Center for Excellence in Primary Care, codifies enablers and attributes of high-performing primary care that can guide self-improvement work, towards operationalization of the Institute for Healthcare Improvement’s Quadruple Aim (Bodenheimer et al. 2014). Key functions outlined by the framework, such as empanelment, team-based care, population management and quality improvement (related to performance dimensions of access, comprehensiveness, coordination and continuity) require comprehensive, accurate and real-time measures of biopsychosocial complexity, at both client and population-levels (Starfield 1998; Friedman et al. 2005).

VCH currently uses the “*AMPS*” survey tool to measure and assess the complexity profile of primary care clients (“*Attachment, Medical conditions, Psychological/mental health/addictions challenges and Socio*-*economic status*”) (Shukor et al. 2018). The AMPS tool was adapted and designed based on the Minnesota Complexity Assessment Method (MCAM, derived from the Dutch INTERMED tool), and was integrated into the Health Authority’s EMR, providing a standard that enables providers to assess patient complexity, guide attachment to providers, and to develop individualized care plans (Shukor et al. 2018; De Jonge et al. 2001; De Jonge et al. 2005; Huyse et al. 1999, 2001; Stiefel et al. 1999; Pratt et al. 2015).

The AMPS and other suite of biopsychosocial complexity survey tools derived from the INTERMED and the rich Dutch tradition in biopsychosocial medicine are theoretically sophisticated and robust, enabling key person-oriented functions related to provider–patient communication and care planning (De Jonge et al. 2001; Boenink and Huyse 1997; Querido 1968a, b; Community Mental Health in the Netherlands 1968). Their limitations are, however, significant and generic, inherent to inefficiencies and subjectivity of questionnaire methodology in general. Their respective functionalities (particularly at the individual patient care level) could be strengthened if their use is complemented with person-oriented knowledge synthesized from other existing databases.

### Measuring and Assessing Biopsychosocial Complexity: A Multi-sourced Data Analytics Approach

The inability to effectively and efficiently characterize and model biopsychosocial profiles at population and client levels had been a long-standing, crucial and unaddressed barrier hindering the design, organization, management and delivery of VCH’s CHC services (Shukor et al. 2018). A VCH team involved in the redesign of VCH’s community-based mental health, substance use and primary care services hypothesized that the ability to effectively and efficiently access, synthesize and analyze cross-cutting databases to generate *real*-*time person*-*oriented biopsychosocial complexity profiles* would enable CHCs to operationalize the fundamental building blocks of primary care. It is important to clarify that “real time” refers to the processes of complexity profile score calculation, and not the frequency of how often source data (i.e. stored within databases) are updated.

Leveraging their professional backgrounds in medicine, health informatics, software programming, health services research, organizational management and engineering, the team designed and developed a software tool (the ‘*VCAT*-*Complexity Module, or ‘VCAT*-*CM’*) that leverages the power of linking existing databases, to calculate and generate *person*-*oriented biopsychosocial complexity profiles*. The VCAT-CM was initially designed and developed to enable specific practical functions at organizational and clinical governance levels related to: (1) assessing whether groups and/or individuals meet the CHC’s mandate, (2) optimizing and balancing the client panels of health care providers, (3) optimizing the composition and organization of multidisciplinary teams, (4) assessing workload content, (5) enabling recognition of client needs and the tailoring of individualized care plans, and (6) monitoring and assessment of changes in individual and population complexity profiles.

This paper describes and discusses the VCAT-CM’s conceptualization, design and development process, and the results and face validity of its complexity score outputs. The potential implications of the study’s findings on the development of the VCAT-CM and the operationalization of the building blocks of primary care framework are then highlighted.

## Methods

Thematic content analysis of the Vancouver Community Primary Care Mandate Statement (“Appendix”) was used to develop and define the domains of VCAT-CM’s conceptual framework.

Administrative and clinical data sources (i.e. record databases) that could be leveraged to populate the domains with content (i.e. scoring variables) were identified according to their relevance, availability and face value. Record data elements that could be used to calculate a complexity score for each domain were identified and selected in accordance to their availability, validity and discriminatory power.

The complexity scoring calculation was developed using an exploratory approach, leveraging the VCAT-CM team’s professional and clinical experience, face validity and specifications of tool and data standards. Complexity scores were calculated for each domain (“Q-scores”) using a Likert-type scale (0–4). Q-scores were used to calculate a Composite Complexity Score (CCS). This was done by dividing each Q-score by 4, resulting in an adjusted probability value (i.e. a *p* value between 0 and 1). The CCS is calculated using the root sum squared method, yielding composite patient complexity values ranging between 0 and 3.

$$ Composite \,Complexity\, Score = \sqrt {Q1^{2} + Q2^{2} + Q3^{2} + Q4^{2} + Q5^{2} + Q6^{2} + Q7^{2} + Q8^{2} + Q9^{2} } $$

To be fit for purpose, the domains were weighted in accordance to CHC staff perceptions of each domain’s relative importance in determining patient complexity. This was done by developing and administering a five-point Likert scale email survey to all CHC staff (administrative and multidisciplinary care, including General Practitioners (GPs), Nurse Practitioners (NPs), Registered Nurses (RNs), Licensed Practical Nurses (LPNs), Social Workers (SWs), Clinical Assistants (CAs) and Clinical Services Coordinators (CSCs)).

The survey strategy stemmed from discussions with CHC staff, which highlighted that not all nine domains of the mandate held the same importance among staff when trying to determine is the complexity profile of a client. It was agreed that the best strategy to address this issue was to create and administer a survey to assess the importance of each mandate domain at the CHC level.

The importance of each domain was grouped and ranked into two categories: (1) Very Important or Important, and (2) Not Important, Slightly Important or Moderately Important. To achieve a range of composite scores between 0 and 3, a weighting factor of 1.20 (+ 20%) was assigned to domains rated in the first category, whereas a weighting factor of 0.75 (− 25%) was assigned to the domains rated in the second category. This methodological approach was also agreed upon by the CHC team. VCAT-CM outputs always render and report both weighted and unweighted composite and domain-specific scores.

The VCAT software’s ability to generate a unified Virtual Patient Record (VPR), in conjunction with the software’s core analytic engine, enabled the calculation and reporting of weighted, unweighted, partial and composite complexity scores for a VCH owned and operated CHC.

The face validity of the VCAT-CM’s outputs (i.e. weighted and unweighted partial and composite scores) was assessed by two of the CHC’s physicians, one of whom is the CHC’s medical director. One physician assessed the face validity of the scores for a small sample, whereas the other physician assessed the face validity of scores for their entire case load of patients, as well as the aggregate distribution of composite scores for the CHC.

The authors declare no known conflicts of interest, and certify their responsibility for the manuscript. A human subject committee (institutional review board) was not required for review or approval of this study, which is in compliance with the Academic Medical Center (AMC, University of Amsterdam) standards and regulations.

## Results

### VCAT-CM Conceptual Domains

Thematic content analysis of the mandate statement resulted in the following nine domains, which serve as the conceptual framework of the VCAT-CM (Table 1).

**Table 1:** VCAT-CM conceptual domains

Domain	Definition
Q1: Attachment	Clients unattached or poorly attached despite need for primary care
Q2: Service density	Clients attached to primary care providers but experiencing a period of functional instability that are challenging to manage within a Fee for Service (FFS) practice. These clients use multiple (and poorly coordinated) health and social care program area services, coupled with access challenges (manifested by no-shows). Intent of CHC engagement would be to stabilize the client, rationalize services, and support eventual transition back to community (FFS) primary care provider where possible
Q3: Social and environmental factors	Clients with multiple social barriers such as housing instability, poverty etc. that impact on the ability to maintain a connection to care
Q4: Psychosocial factors	Clients with marked difficulties in accessing the fee-for-service health care system due to significant cognitive, behavioral and/or functional impairment
Q5: Relationships	Inability to maintain lasting personal or professional relationships
Q6: Activities of daily living (ADLs)	Clients with marked difficulties with activities of daily living without access to appropriate supports
Q7: Medical complexity	Medically complex conditions presenting with chronic disease, concurrent disorders or communicable diseases (i.e. diabetes, hepatitis, HIV, mental health issues, substance misuse) that are untreated or uncontrolled
Q8: Acute (hospital) utilization	High emergency department use for issues that could be addressed in the primary care setting and/or frequent acute care admission/readmission rates
Q9: Risk of harm to self or others	Risk of causing harm to self or others

**Fig. 1:**
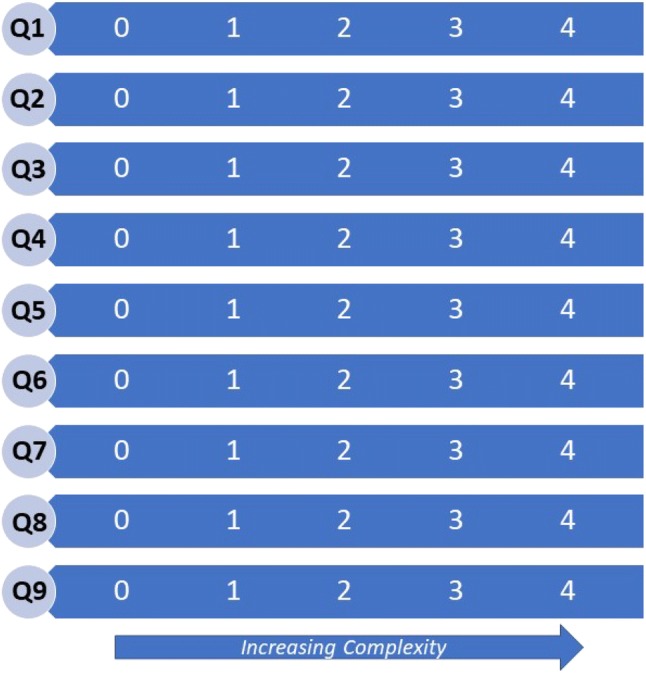
Biopsychosocial complexity profile comprised of nine domains (Qs)

Patient complexity is conceptualized as a multidimensional person-oriented profile comprised of the nine domains, which are measured as vectors (i.e. having magnitude and direction). Arrayed in parallel, the vectors form a profile of complexity, with each domain receiving a partial complexity score (Q-score) designed on a 0–4 Likert-type (0–4) scale (Fig. 1).

### Complexity Calculation

Table 2 below outlines: (1) the data sources and elements used to derive Q-scores; (2) the rationale for use of each data element; (3) the Q-score calculation system and (4) the main rationale behind the Q-score calculation methodology.

**Table 2:** VCAT-CM Complexity Score calculation

Domain	Data sources and elements	Rationale (selection of data sources and elements)	Q-scoring system	Rationale (Q-scoring system)
Q1	*IntraHealth Profile EMR* Encounters *PARIS EMR* Encounters	Very reliable information and readily available. In accordance with definitions of attachment/un-attachment from a variety of sources (i.e. British Columbia Ministry of Health (MoH), DTES 2^nd^ generation services [a new model of care that will give residents of the DTES better access to coordinated, consistent health care; it brings together existing programs and services so clients get the care they need at one location], Primary Care)	Score = 0: clients with 4 + visits in past 18 months (equally dispersed) Score = 1: clients with 3 visits in past 18 months Score = 2 : clients with 2 visits in past 18 months Score = 3 : clients with 1 visit in past 18 months Score = 4 : clients with no visits in past 18 months	Included in the calculation were the clients who had at least 1 encounter in the past 18 months. Encounters equally dispersed over the period of 18 months were considered (i.e. encounters which were less than a week apart were collapsed into a single encounter). The higher the number of encounters, the lower the attachment score
Q2	*IntraHealth Profile EMR* Encounters *PARIS EMR* Encounters Referrals to services	Very reliable information and readily available. In accordance with definitions of attachment/un-attachment from a variety of sources (MoH, DTES 2^nd^ generation services, Primary Care)	Score = 0 : clients seen in one service/program Score = 1 : clients seen in two services/programs Score = 2: clients seen in three services/programs Score = 3: clients seen in four services/programs Score = 4: clients seen in more than four services/programs If # of NSBA (no show with booked appointment) in past 18 months > 10 elevate score by 1	The higher the number of simultaneously open referrals a client has, the higher the complexity score. If the client has 10 + no shows with booked appointment in the past 18 months the complexity score is elevated by 1
Q3	*PARIS EMR* Latest HoNOS Assessment: question 11 for Housing instability and Question 12 for problems with occupation and activities *IntraHealth Profile EMR* Persons With Disabilities (PWD) forms Social History (SHX) codes	Validity of the HoNOS assessment and its sensitivity to small changes in scores	Score = 0: a score of 0 for HoNOS Question 11 or 12 Score = 1: a score of 1 for HoNOS Question 11 or 12 Score = 2: a score of 2 for HoNOS Question 11 or 12 Score = 3: a score of 3 for HoNOS Question 11 or 12 Score = 4: a score of 4 for HoNOS Question 11 or 12 AND/OR the client has PWD forms AND/OR SHX problems recorded	The higher the score on the HoNOS question, the higher the complexity score. If PWD form present and social history codes present the score is elevated
Q4	*PARISProfile EMR* Latest HoNOS Assessment (Q4 for cognitive, Q1 and Q8 for behavioral, Q5 for functional impairment)	Validity of the HoNOS assessment and its sensitivity to small changes in scores	Score = 0: a score of 0 for HoNOS Question 1 AND/OR Question 4 AND/OR Question 5 AND/OR Question 8 Score = 1: a score of 1 for HoNOS Question 1 AND/OR Question 4 AND/OR Question 5 AND/OR Question 8 Score = 2: a score of 2 for HoNOS Question 1 AND/OR Question 4 AND/OR Question 5 AND/OR Question 8 Score = 3: a score of 3 for HoNOS Question 1 AND/OR Question 4 AND/OR Question 5 AND/OR Question 8 Score = 4: a score of 4 for HoNOS Question 1 AND/OR Question 4 AND/OR Question 5 AND/OR Question 8	The higher the score on the HoNOS question, the higher the complexity score
Q5	*PARIS EMR* Latest HoNOS Assessment (Q9, Q11 and Q12) *IntraHealth Profile EMR* SHX codes	Validity of the HoNOS assessment and its sensitivity to small changes in scores	Score = 0: a score of 0 for HoNOS Question 9 AND/OR HoNOS Question 11 AND/OR Question 12 Score = 1: a score of 1 for HoNOS Question 9 AND/OR HoNOS Question 11 AND/OR Question 12 Score = 2: a score of 2 for HoNOS Question 9 AND/OR HoNOS Question 11 AND/OR Question 12 Score = 3: a score of 3 for HoNOS Question 9 AND/OR HoNOS Question 11 AND/OR Question 12 Score = 4: a score of 4 for HoNOS Question 9 AND/OR HoNOS Question 11 AND/OR Question 12 AND/OR the client has SHX problems recorded	The higher the score on the HoNOS question, the higher the complexity score. If social history codes present then the score is elevated
Q6	*PARIS EMR* InterRAI-MDS assessment in Home Health (MAPLE scores, CAPS) Occupational Therapy (OT)/Physiotherapy (PT) assessments for mobility Latest HoNOS Assessment (Q5 for physical illness and disability, Q10 for activities of daily living, Q11 for housing and Q12 for occupation and activities)	Validity of the HoNOS assessment and its sensitivity to small changes in scores, validity of InterRAI tool	Score = 0: a score of 0 for HoNOS Question 5 AND/OR Question 10 AND/OR Question 11 AND/OR Question 12 Score = 1: a score of 1 for HoNOS Question 5 AND/OR Question 10 AND/OR Question 11 AND/OR Question 12 Score = 2: a score of 2 for HoNOS Question 5 AND/OR Question 10 AND/OR Question 11 AND/OR Question 12 Score = 3: a score of 3 for HoNOS Question 5 AND/OR Question 10 AND/OR Question 11 AND/OR Question 12 Score = 4: a score of 4 for HoNOS Question 5 AND/OR Question 10 AND/OR Question 11 AND/OR Question 12 AND/OR INTERRAI-MDS Ax AND/OR Transfer/Bed Mobility Ax (PARIS)	The higher the score on the HoNOS question, the higher the complexity score. If InterRAI-MDS assessment and/or OT/PT assessments for mobility present then the score is elevated
Q7	*IntraHealth Profile EMR* Problem List Medications (EMR) PSW forms SHX codes *PARIS EMR* Latest HoNOS Assessment (Q6, Q7, Q8 for mental health issues, Q3 for substance misuse)	Validity of the HoNOS assessment and its sensitivity to small changes in scores	Score = 0: no diagnosis recorded in the problem list Score = 1: 4 + diagnoses recorded in the problem list Score = 2: any SU/MH diagnosis recorded in the problem list AND client not on Extended Leave Score = 3: 2 + diagnoses recorded in the problem list AND any SU/MH diagnosis (Schizophrenia) Score = 4: Complex Care Diagnostic Codes (according to General Practice Services Committee—GPSC, Jan 2018) OR 6 + diagnoses recorded in the problem list OR (any SU/MH diagnosis AND client on Extended Leave) Or a Chronic Neurodegenerative Disorder or a score of 4 on any question in the last HoNOS assessment If more than 5 dx elevate score by 1 If BP > 140/90 elevate score by 0.5 If BMI > 25 elevate score by 0.25 If BMI > 30 elevate score by 0.5 If BMI > 35 elevate score by 0.75	The higher the number of chronic conditions, the higher the score. If MHSU conditions present the score is elevated. If there exist combinations of chronic conditions (according to GPSC) the score is elevated even more. If BMI > 25 or BP > 140/90 score is again elevated
Q8	*EDMart and AcuteMart* ED visits by CTAS LOS (acute admissions)	Accurate information, face validity, availability of data	Score = 0: Hospitalization Complexity Score of 0 Score = 1: Hospitalization Complexity Score between 1 and 24 Score = 2: Hospitalization Complexity Score between 15 and 25 Score = 3: Hospitalization Complexity Score between 25 and 50 Score = 4: Hospitalization Complexity Score > 50	Calculation based on inverse CTAS score and Length of Stay in hospital. The higher the combined score, the higher the complexity score
Q9	*IntraHealth Profile EMR* Alerts (violence) PHQ Extended leave *PARIS EMR* Extended Leave Alerts (violence) HoNOS Assessment (Q1 and Q2)	Validity of the HoNOS assessment and its sensitivity to small changes in scores	Score = 0: a score of 0 for HoNOS Question 1 AND/OR HoNOS Question 2 Score = 1: a score of 1 for HoNOS Question 1 AND/OR HoNOS Question 2 Score = 2: a score of 2 for HoNOS Question 1 AND/OR HoNOS Question 2 Score = 3: a score of 3 for HoNOS Question 1 AND/OR HoNOS Question 2 Score = 4: a score of 4 for HoNOS Question 1 AND/OR HoNOS Question 2 AND/OR the client has violence alerts recorded AND/OR client is on Extended Leave AND/OR client has a score greater than 9 on the latest PHQ9	The higher the score on the HoNOS question, the higher the complexity score. If violence alerts present or client on extended leave, the score was elevated

### Complexity Domain Weighting

**Chart 1:**
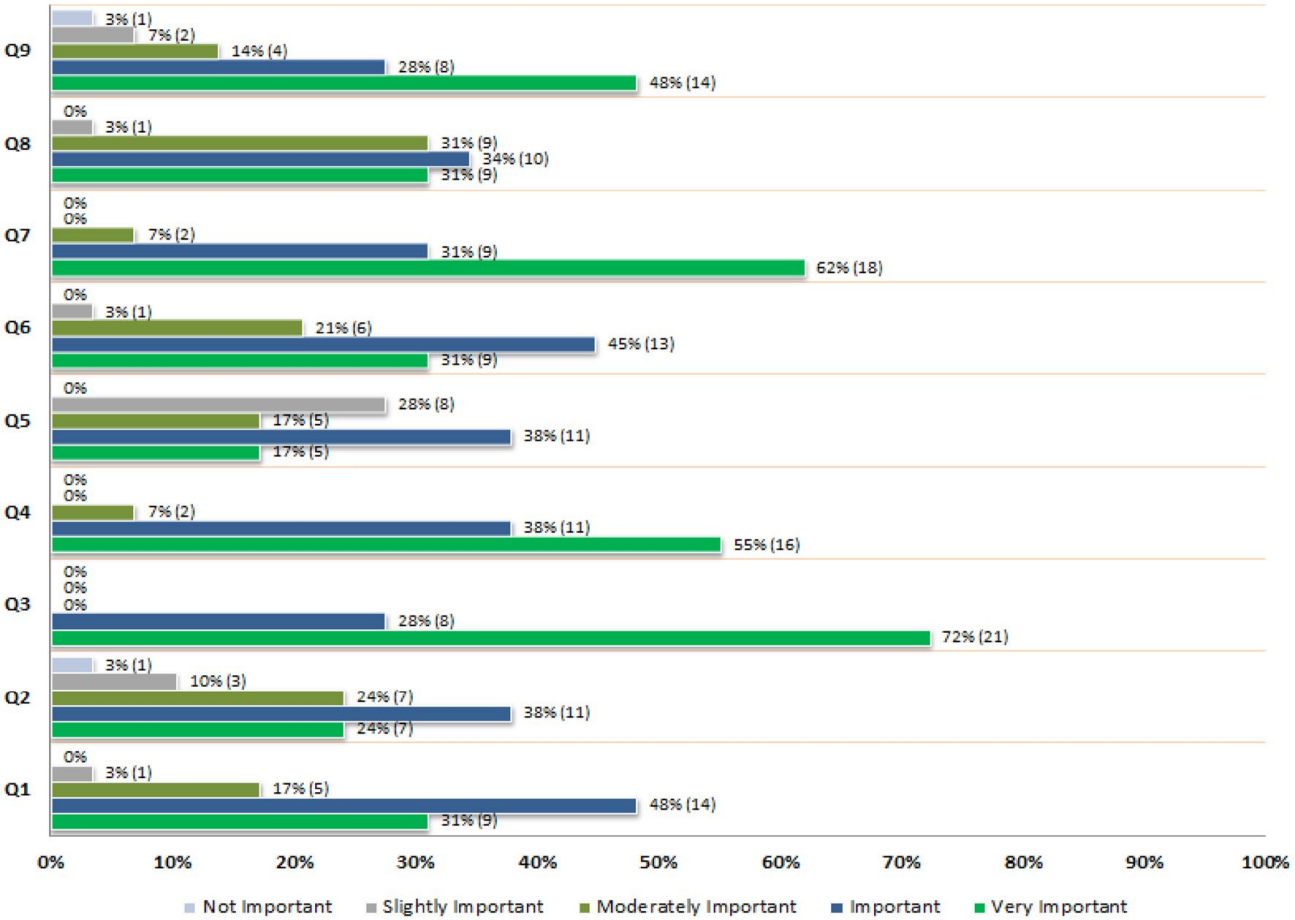
Perceived importance of complexity domains

Seventy-five percent of CHC primary care staff responded to the five-point Likert scale survey, representing a wide spectrum of multidisciplinary clinical and administrative staff (n = 29; comprised of 10 GP/NPs, 8 RN/LPNs, 3 SWs, 3 CA/CSCs and 5 “other staff”). The breakdown of the perceived importance of each complexity domain is presented in Chart 1.

**Chart 2:**
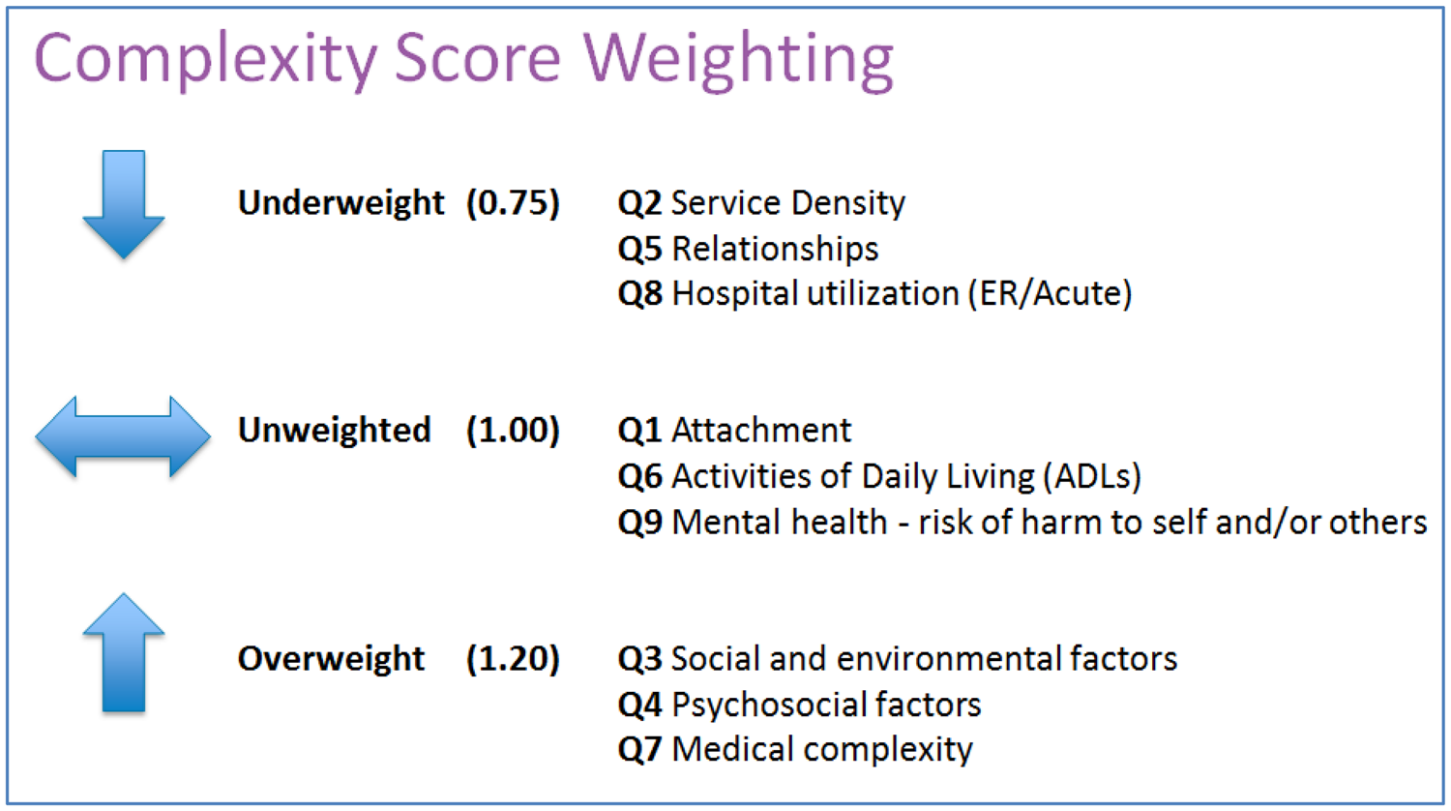
Weighting of complexity domains

Social and environmental factors (Q3), psychosocial factors (Q4) and medical complexity factors (Q7) were therefore over-weighted, with a factor of 1.20. Attachment (Q1), activities of daily living/ADLs (Q6) and mental health/risk of harm to self and/or others (Q9) were weighted with a factor of 1.00 (Chart 2). Service density (Q2), relationships (Q5) and hospital utilization (Q8) were under-weighted, with a factor of 0.75.

### VCAT-CM Outputs

**Chart 3:**
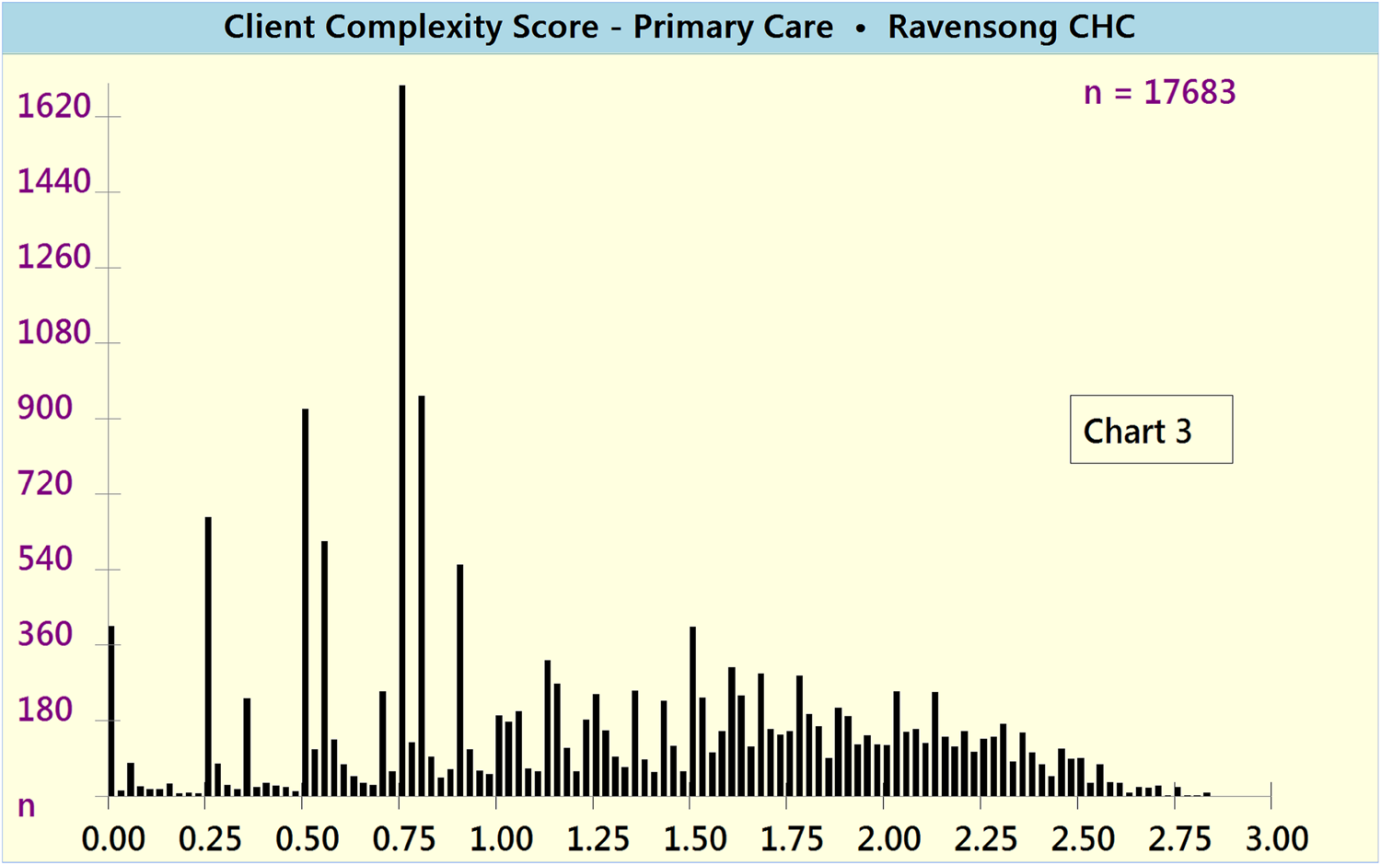
Unweighted Composite Complexity Scores (CCS)

**Chart 4:**
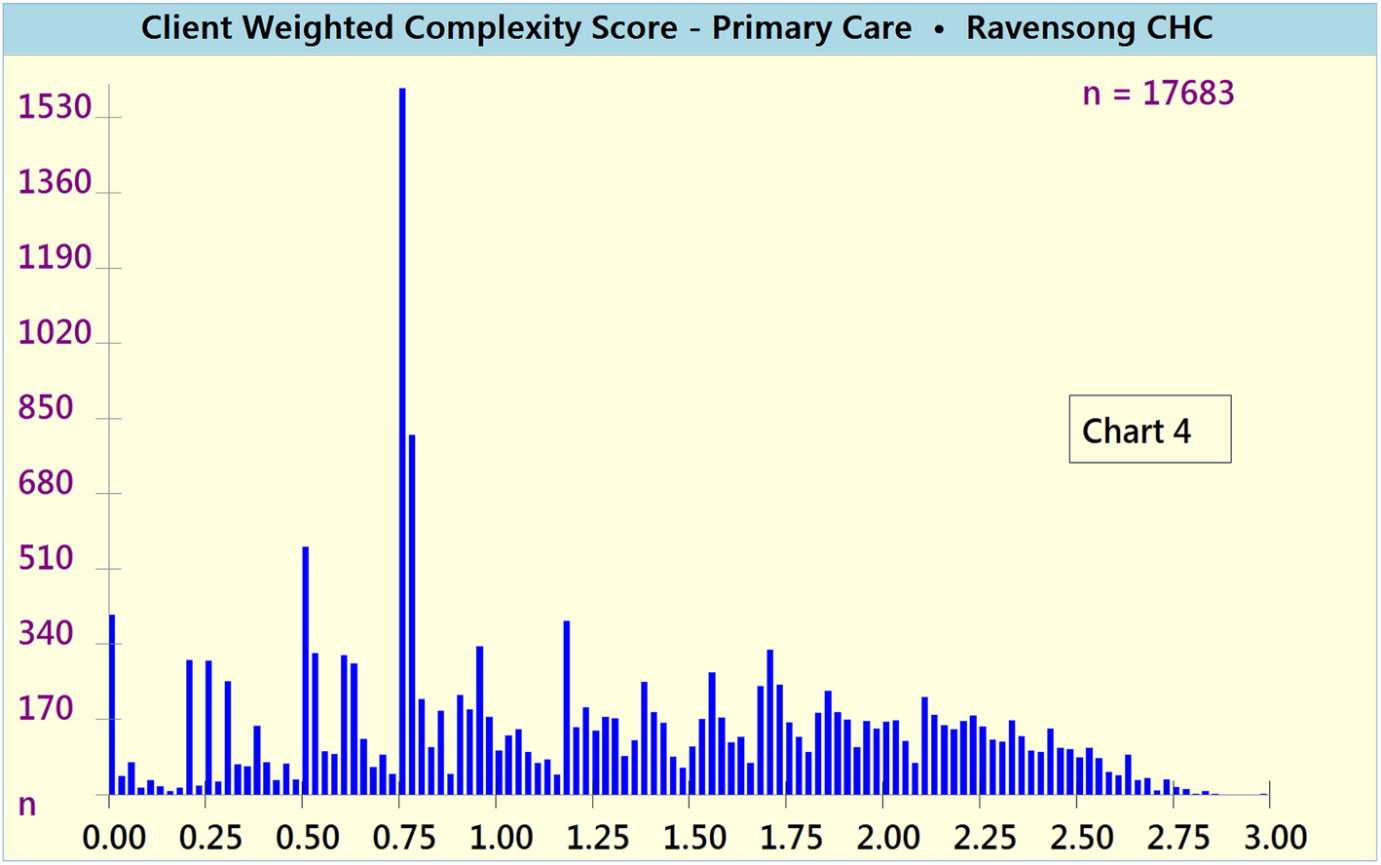
Weighted Composite Complexity Scores (CCS)
- Unweighted and weighted Domain-specific disaggregated CCS [Charts 5 and 6, respectively]

The VCAT-CM was used to calculate and report the following complexity scores for VCH’s Raven Song CHC:

- Unweighted and weighted Composite Complexity Scores (CCS) [Charts 3 and 4, respectively]

**Chart 5:**
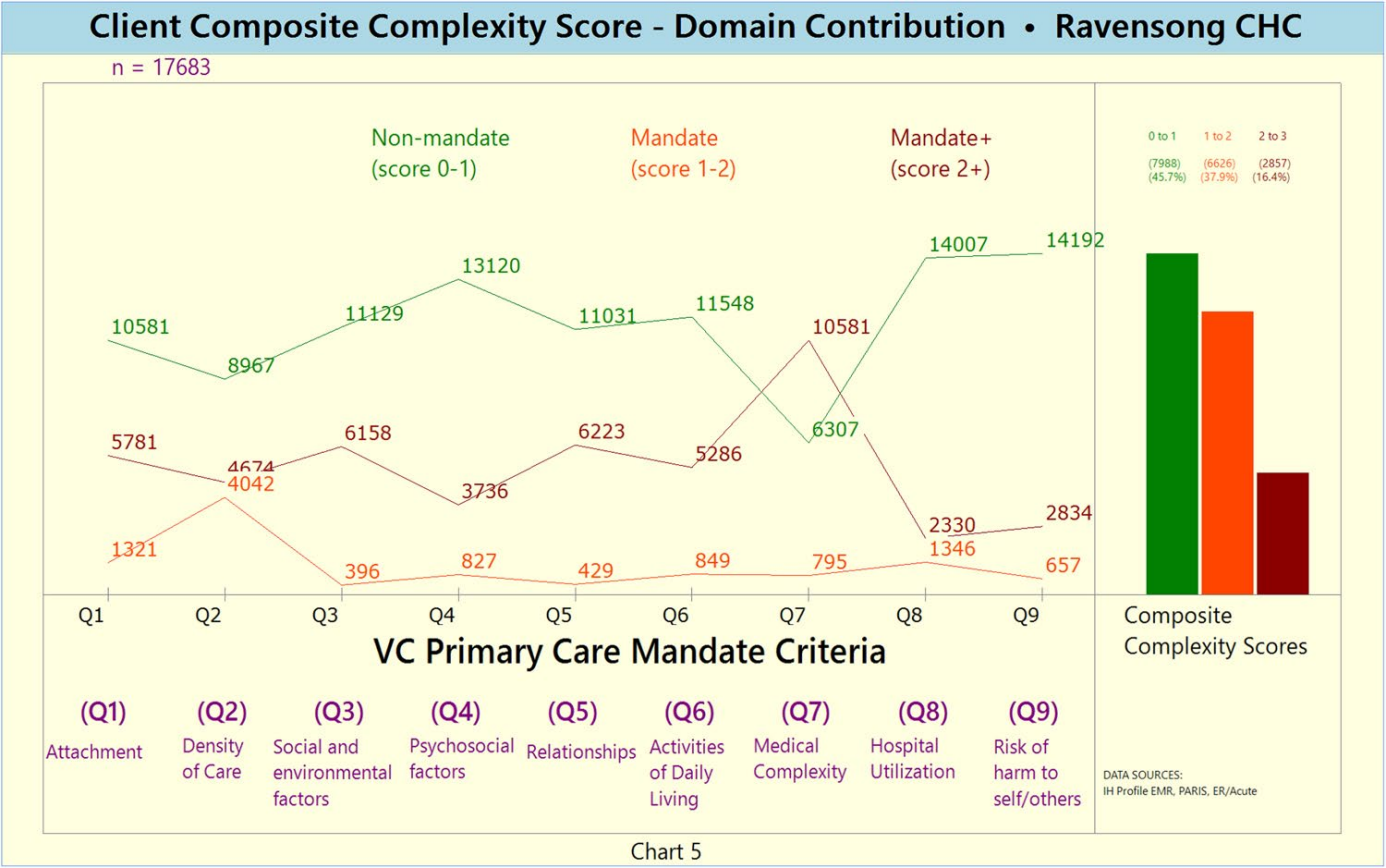
Unweighted Domain-specific disaggregated Composite Complexity Scores (CCS)

**Chart 6:**
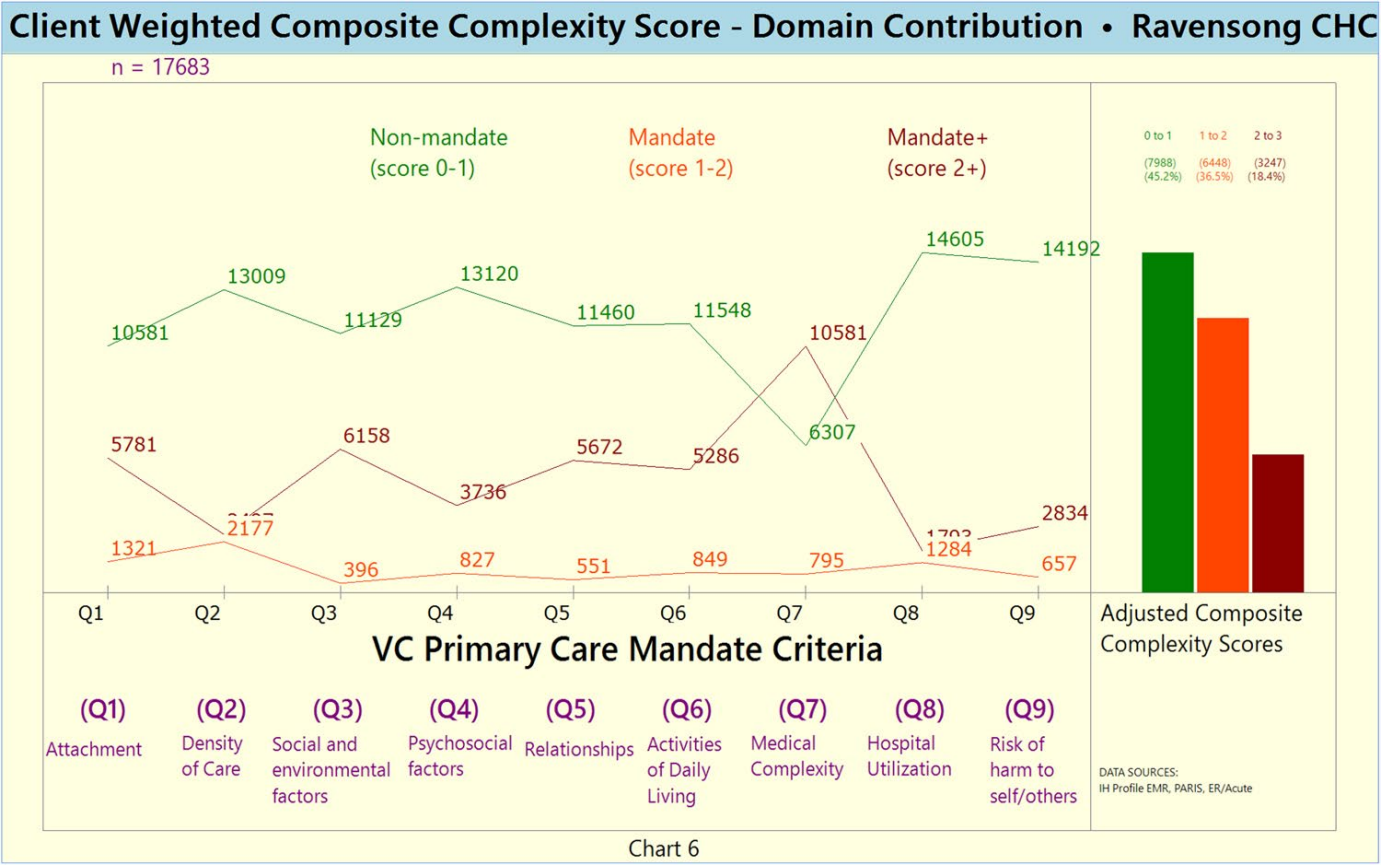
Weighted Domain-specific disaggregated Composite Complexity Scores (CCS)

**Chart 7:**
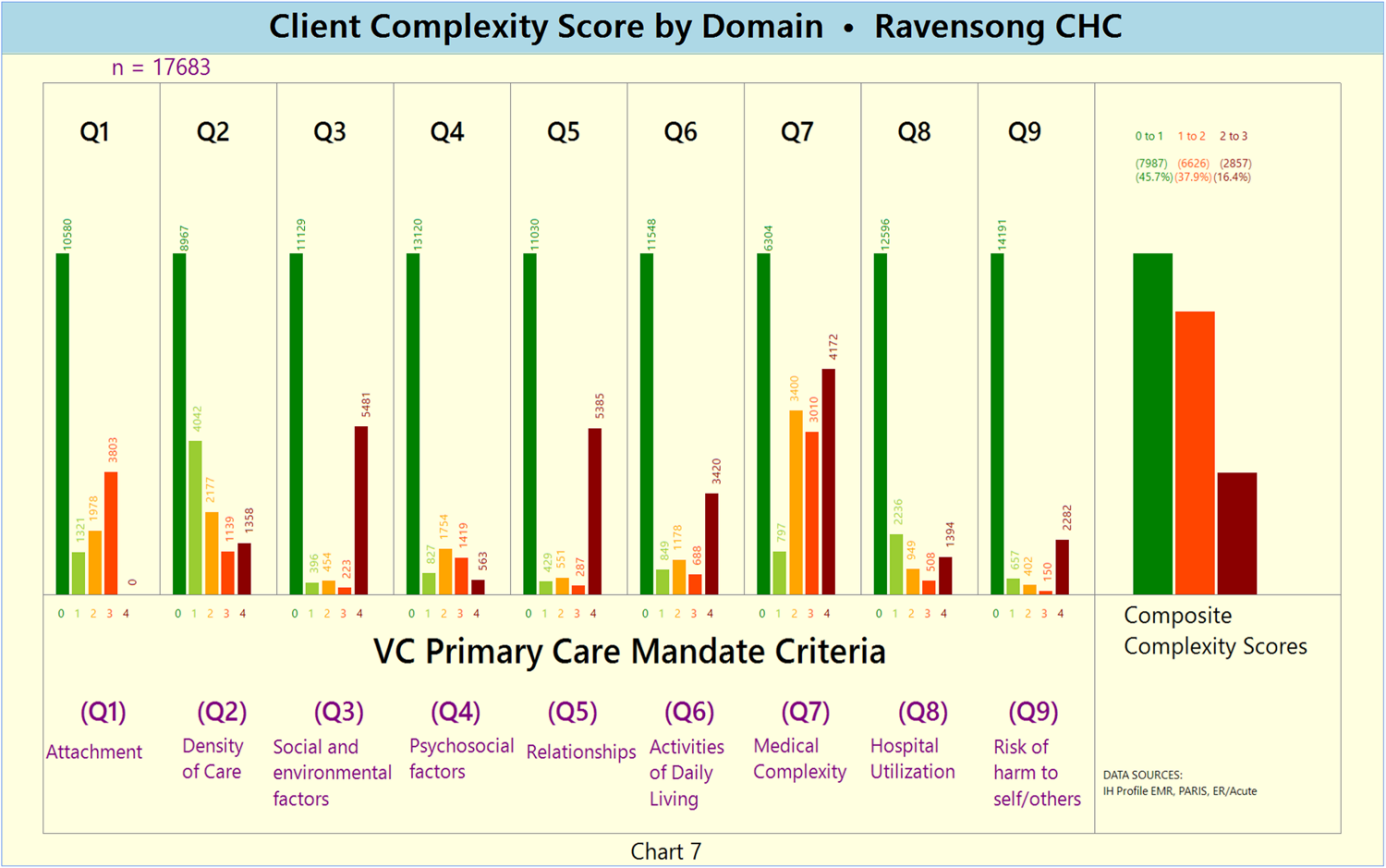
Unweighted Domain-specific Complexity Score

**Chart 8:**
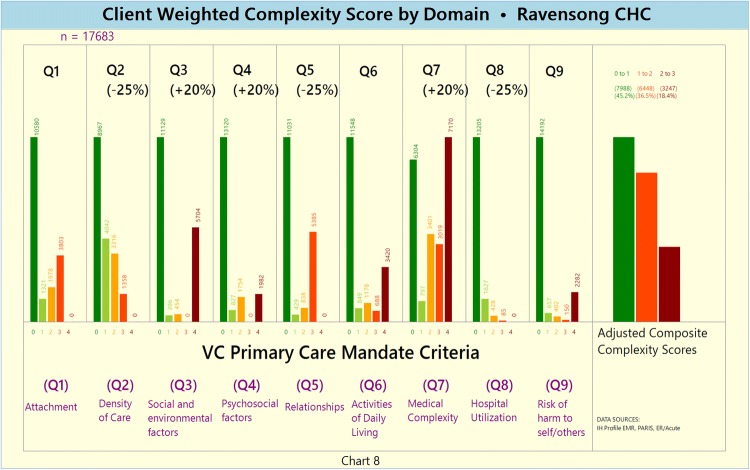
Weighted Domain-specific Complexity Score

  - Disaggregated according to the following complexity score intervals: Score 0–1, Score 1–2, and Score 2 + .
- Unweighted and weighted domain-specific complexity score (Charts 7 and 8, respectively)

The composite complexity scores (CCS, weighted and unweighted) bring to the light the high absolute numbers and proportions of clients who potentially do not meet the mandate of VCH CHCs (i.e. low CCS ranging from 0 to 1), along with the high absolute numbers and proportions of highly complex (i.e. CCS 2–3) clients who potentially meet mandate specifications.

The disaggregated domain-specific complexity scores highlight that domains Q2 (service density), Q6 (ADLs) and Q7 (medical complexity) are characterized by relatively high proportions of complex clients.

**Chart 9:**
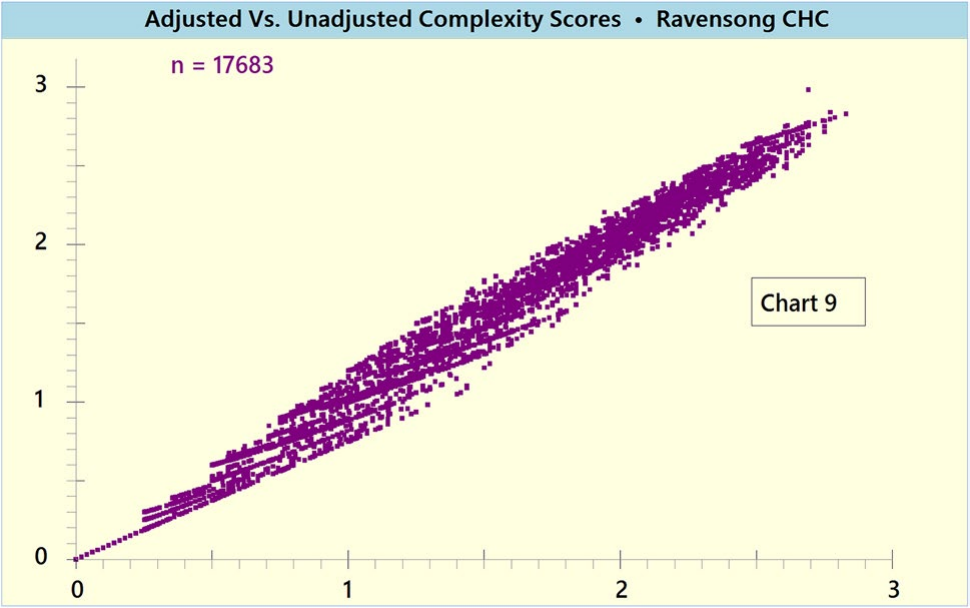
Delta between weighted and unweighted Composite Complexity Scores (CCS)

Chart 9 presents the delta (i.e. difference) between the weighted and unweighted composite complexity scores. The scatterplot reflects the low to moderate impact the weighting factors had.

### Face Validity

VCAT-CM outputs (i.e. weighted and unweighted composite and domain-specific complexity scores) manifested strong face validity. On a CHC client population level, both unweighted and weighted results (Charts 3 and 4, respectively) were perceived by the CHC’s medical director to accurately reflect the distribution of client complexity.

At an individual client level, two GPs perceived that Composite Complexity Scores (CCSs) provided realistic, accurate and updated depictions of their respective clients’ complexity profiles. Q-scores were perceived to accurately depict the combinations of complexity that clients manifested. Complexity scores were perceived to have good discriminatory power, in that they enable differentiation of patients in accordance to their unique contexts.

## Discussion

This paper describes the conceptualization, development and testing of a novel software tool (the VCAT-CM) that can calculate and report real-time person-oriented biopsychosocial complexity profiles, using readily available data sources.

The VCAT-CM conceptualizes complexity as a *profile* comprised of nine domains, all of which are vectors. Arrayed in parallel, they form a profile of complexity which aligns to Starfield’s proposed approach for measurement of outcomes, which calls for a scheme that is based upon the development of a profile rather than simply a singular index (Starfield 2005). The profile approach is also operationalized by the Dutch Self Sufficiency Matrix (SSM), INTERMED, Patient Centered Assessment Method (PCAM), MCAM and AMPS tools (Shukor et al. 2018; De Jonge et al. 2001; Huyse et al. 2001; Pratt et al. 2015; Lauriks et al. 2014). There are also interesting parallels between the VCAT-CM and Safford’s ‘vector’ model of complexity, which depicts each determinant of complexity as a vector influencing the direction and magnitude of a patient’s complexity (Safford et al. 2007).

The tool’s conceptual domains were derived using an inductive, participatory and evidence-based developmental approach. The tool was created by and for a team requiring effective, efficient and practical mechanisms to accurately measure and assess the biopsychosocial complexity profiles of presenting patients. Such profiles are required to enable ongoing VCH functions relating to operationalization of the fundamental building blocks of primary care, such as empanelment, team-based care, data-driven improvement and population management (Shukor et al. 2018). The VCAT-CM’s design is therefore attuned to the developmental state (i.e. contextual reality) of the system, only leveraging existing and necessary resources, and delivering outputs that are practical and actionable.

The Vancouver Community Primary Care Mandate Statement conceptually underpins the content of the VCAT-CM (“Appendix”). The content of the mandate was developed over a period of 3 years, using a reflexive process involving extensive consultation with VCH primary care directors, managers and front line multidisciplinary clinical and administrative staff. This inductive, inclusive and iterative approach resulted in rich content that comprehensively depicts multi-disciplinary and multi-professional perceptions of the biopsychosocial characteristics, needs and service utilization patterns of the target sub-populations being served, coupled with perceptions of commensurate requirements of primary care service delivery models. The key strength of this approach is that it enabled a robust synthesis of varied perspectives of the biopsychosocial realities and needs of the complex sub-populations being served. In essence, this approach aligns with Starfield’s vision of “*balancing health needs, services and technology*” (Starfield 1998).

Thematic content analysis of the mandate resulted in the synthesis of the VCAT-CM’s nine domains, which aligns theoretically robust complexity frameworks (e.g. Schaink et al. 2012), and cross-maps with many of the specific dimensions of other biopsychosocial complexity tools such as the Self-Sufficiency Matrix (SSM), INTERMED tool, Minnesota Complexity Assessment Method (MCAM), and AMPS tool (Shukor et al. 2018; De Jonge et al. 2001; Pratt et al. 2015; Lauriks et al. 2014; Safford et al. 2007; Schaink et al. 2012; Loeb et al. 2015; Fassaert et al. 2014).

The content of each of the nine complexity domains are comprised of discrete data elements populating readily available administrative and clinical databases. Making optimal use of available, relevant and valid data is a key underpinning principle of the VCAT-CM. All data elements reflect or bear some hypothetical relationship to the processes, outputs or outcomes of care. Where possible, data elements are derived from validated clinical assessment tools, such as the HoNOS and RAI-MDS (Pirkis et al. 2005; Carpenter 2006; Poss et al. 2008). Other data elements are subject to record data entry organizational standards (e.g. primary care EMR data), which are the focus of Canadian provincial quality improvement efforts (BC General Practice Services Committee 2018; Primary Care Practice Reports—Health Quality Ontario (HQO) 2018; Health Data Coalition 2019; Canadian Primary Care Sentinel Surveillance Network 2018). Furthermore, the HoNOS, which is answered on an item-specific anchored 4-point scale (with higher scores indicating more problems) aligned well with the VCAT-CM’s scoring system (Pirkis et al. 2005). The content of the scoring system will undergo further refinement using a developmental evaluation approach, as well as a rigorous process of content validation.

To ensure the VCAT-CM is fit for purpose at the CHC test site, the complexity domains were weighted using survey results from 29 CHC primary care staff, representing a wide spectrum of multidisciplinary clinical and administrative staff. Virtually none of the VCAT-CM’s domains were statistically perceived to be “not important”, which provides initial and cursory reassurance of their validity.

As may be expected from a CHC setting, social and environmental factors (Q3), psychosocial factors (Q4) and medical complexity factors (Q7) were perceived to be the most important factors informing the development of a complexity profile. What is particularly interesting is that CHC staff put less importance on hospital utilization, which is focused upon by policymakers, organizational leaders, performance management stakeholders and health services researchers (Van den Heede and Van de Voorde 2016; Sutherland and Crump 2013). The unweighted complexity output for domain Q8 (acute/hospital utilization) preliminarily reinforces the validity of the CHC staff’s perception.

The unweighted output of domain Q2 (service density), however, points to the fact that service density is perhaps one of the most important and influential of the nine complexity domains—something not initially perceived by the CHC’s staff (as it was underweighted). The Q2 domain’s output may potentially be interpreted to support the narrative that CHC clients (e.g. presenting with histories of challenging patient–provider relationships) may be over-serviced but under-served (Shukor et al. 2018).

The weighting exercise demonstrated the ease of adjusting complexity domain weightings to suit local contexts, values and perceptions—a key strength of the VCAT-CM. It is important to note that the VCAT-CM will always report both weighted and unweighted complexity scores, since values imparted by stakeholders will vary by context and time (Starfield 2005).

On a CHC client population level, both unweighted and weighted composite complexity scores (Charts 3 and 4, respectively) were perceived by the CHC’s medical director to accurately reflect the distribution of client complexity. The significant number and proportion of low complexity (i.e. CCS = 0–1) clients is potentially due to the fact that the CHC also operates a separate youth clinic (mainly offering public health and sexual health-related services for youth under 25 years of age), a Trans specialty care program, and a Hepatitis C program. Clients accessing these services and programs are often of low biopsychosocial complexity, yet still use the CHC’s primary care clinic. Many of these clients, however, potentially do not meet the official mandate of VCH primary care (e.g. CCS ≤ 1), and should be attached to community-based primary care clinics, which could appropriately meet their needs. These findings are particularly salient in light of the BC ministry of health’s ‘Primary Care Network’ policy focus on appropriate attachment to primary care (Patient Medical Homes and Primary Care Networks 2018).

The VCAT-CM is therefore being tested to develop and operationalize standard business rules related to appropriate referrals and attachment to community-based primary care. It represents a potentially significant advance that may be complementary to tools such as the SSM, which is used by the Amsterdam Public Health Service to enable decisions related to allocating homeless people to the public mental health care system (Lauriks et al. 2014). It could potentially be helpful in jurisdictions such as Ontario, where it is suspected that CHCs may also be serving low complexity clients already capitated to Family Health Teams (Confidential communication by Ontario health system expert 2018).

VCH is also leveraging the complexity scores to operationalize the fundamental buildings blocks of empanelment and team-based care within the CHCs (Bodenheimer et al. 2014). The VCAT-CM is being used to develop an ‘Empanelment Team Target Compiler’ software tool that calculates optimal configurations of teams based on client complexity scores and health care provider characteristics. A key focus is to ensure that the workload of various CHC team configurations and individual clinicians are appropriate and balanced. The VCAT-CM is therefore enabling the redesign of primary care services, to ensure that service delivery models and multidisciplinary team configurations effectively, efficiently and equitably meet client needs. Efforts are underway to reduce and eventually eliminate the present lag-time of VCAT-CM outputs (i.e. to move from monthly to weekly to real-time analysis and reporting capabilities).

The VCAT’s software innovations are highly relevant to international stakeholders interested in operationalizing the fundamental building blocks of empanelment and team-based care (Shukor et al. 2018; Ghorob and Bodenheimer 2012; Ghorob and Bodenheimer 2015; Wagner et al. 2017; Christiansen et al. 2016; Grumbach and Olayiwola 2015; Teng 2018; Pastore et al. 2013; West et al. 2015). The VCAT’s biopsychosocial approach represents an advance over commonly-used complexity measurement tools such as the Diagnosis Count, Medication Count, Chronic Disease Score (CDS)/RxRisk, Charlson Comorbidity Index (CMI), Johns Hopkins Adjusted Clinical Grouping (ACG) System, Cumulative illness Rating Scale (CIRS), Duke Severity of Illness (DUSOI) Checklist, and Quality and Outcomes Framework (QOF) Score (Park 2016; Huntley et al. 2012). The common limitation of these tools is that they tend to be medical or disease-oriented, with limited ability to incorporate psychosocial or environmental factors (it is important, however, to note that medical issues coded in the problem list such as mental illness and addictions are captured by many of these tools) (Friedman et al. 2005; De Jonge et al. 2001; Pratt et al. 2015; Lewis et al. 2016).

The VCAT-CM may therefore be of use to other jurisdictions such as Ontario, where CHCs and community-based Family Health Teams use the Standardized ACG Morbidity Index (SAMI) (Muldoon et al. 2013). The SAMI represents the ratio of the average ACG for a clinic relative to Ontario’s provincial average ACG, enables the assessment of morbidity patterns and variations, and is used to measure the expected workload in Ontario’s primary care practices (Muldoon et al. 2013).

At an individual client level, two GPs perceived that Composite Complexity Scores (CCSs) provided realistic, accurate and updated depictions of their respective clients’ complexity profiles. Q-scores were perceived to accurately depict the combinations of complexity that clients manifested. Complexity scores were perceived to have good discriminatory power, in that they enable differentiation of patients in accordance to their unique contexts. These are similar characteristics to the PCAM and MCAM, which has been validated for use to enable multidisciplinary care planning functions (Pratt et al. 2015; Maxwell et al. 2011; Maxwell et al. 2018).

It is hypothesized that complexity scores are also sensitive to change, thereby showing worsening or improvement over time, across and between biopsychosocial domains. This has implications on the tool’s use for care functions related to monitoring, along with assessment of health outcomes. This hypothesis will undergo testing, and is depicted through two hypothetical case examples, outlined in Box 1.

**Box 1:**
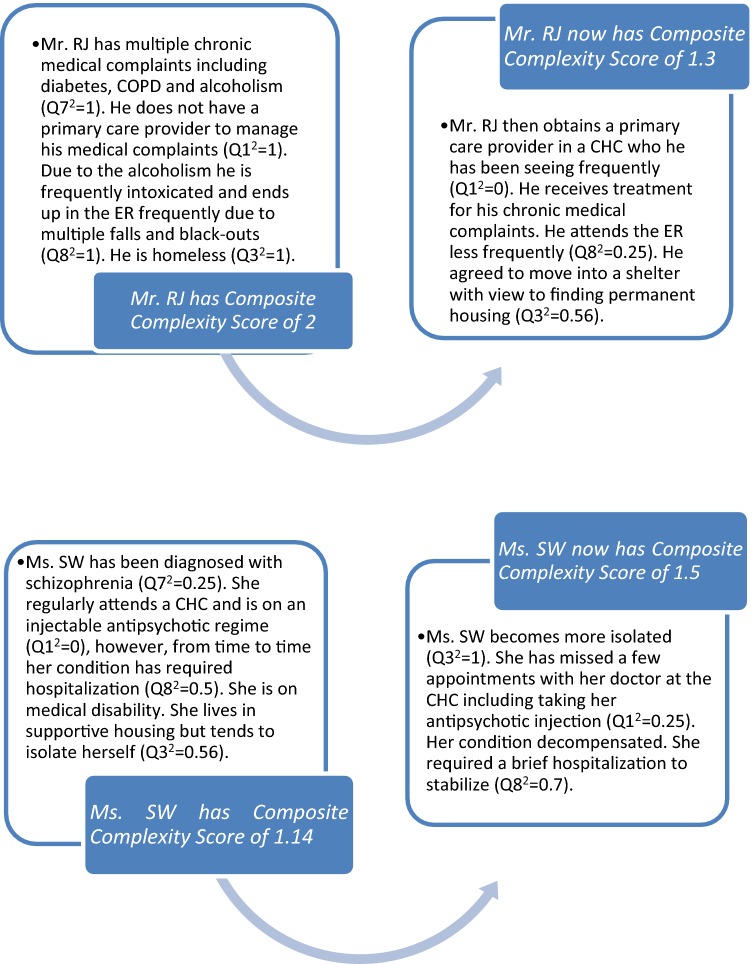
Hypothetical case examples of individual biopsychosocial complexity score transitions

The VCAT-CM continues to develop using an inductive and grass-roots approach, meant to practically respond to the Health Authority’s tactical and strategic challenges at organizational and clinical governance levels. The tool is currently being employed by VCH’s CHCs to operationalize pilot interventions and developmental evaluations related to empanelment, service delivery rationalization (i.e. identifying non-mandate clients, and enabling their attachment to community GPs), optimizing team-based care, care planning and measuring health outcomes. The VCAT-CM is currently undergoing processes of development and validation (i.e. content, construct and criterion validity), to ensure that the software tool is fit for purpose. Results of these validation exercises will be published in subsequent scientific articles.

The complexity algorithm presented in the study was specifically developed for the population served by the VCH, which is a highly complex and marginalized population presenting with multiple co-morbidities and psychosocial issues. The algorithm is also undergoing development and adaptation to meet the needs and realities of other primary care settings and populations, particularly the lower intensity ones (i.e. general community-based primary care) where most of the population served would have complexity scores in the range of 0 to 1. The authors’ proposal to adapt the tool to these settings would integrate other measures into the initial complexity algorithm that would indicate complexity at the lower end of the scale, thereby rendering the algorithm sensitive enough to categorize the individuals within a healthier cohort. This is possible due to the tool’s flexibility, as it contains different subroutines for each of the nine domains that perform calculations of the partial scores These may include age and gender adjusted risk, diet and life style measures (e.g. smoking and alcohol use being integrated into the medical complexity domain (Q7)), addition of numbers of prescriptions, lab tests or assessments within specific periods of time, and addition of numbers of visits to clinics within a specific period of time (as an indication of frequent utilization).

The VCAT-CM is also undergoing development to enable identification and prediction of pre-frail populations who are at risk of frailty. Some of the nine domains used in the complexity algorithm are potentially well suited for predicting frailty. These include poor attachment (missed appointments/cancellations), utilization of clinic drop-ins as opposed to booked appointments (as a marker of instability), medical complexity (number and types of chronic conditions a patient has), service utilization patterns, hospital utilization, social and environmental factors (homelessness or precariously housed), inability to maintain lasting relationships, difficulties with ADLs, disability status, substance use (e.g. Opioid Agonist Treatment) and mental health risks (e.g. behavioral alerts). All these domains, individually or in combination, may potentially be suitable for the prediction of frailty, and are the subject of ongoing investigation.

## Conclusions

Measures of biopsychosocial complexity are required to enable operationalization of the fundamental building blocks of primary care. Initial testing of the VCAT-CM indicates that its outputs have the potential to manifest valid and realistic population and individual level biopsychosocial complexity profiles. The software therefore has implications on the development of key functions related to empanelment, team-based care, population management, performance assessment, quality improvement and funding. The tool’s validity in relation to each of these functions will be gradually and incrementally ascertained within VCH using developmental evaluative approaches. The VCAT-CM potentially fills a significant gap and need in contemporary primary care systems, which to date have been unable to effectively and efficiently leverage existing data to construct person-oriented complexity profiles. These profiles are essential if the domain of primary care is to meaningfully operationalize Starfield’s vision of “*balancing health needs, services and technology*” (Starfield 1998).

## Appendix: VCH Primary Care Mandate Statement (Reviewed August 2017)

VCH Primary Care Mission:

VCH Primary Care provides compassionate, equitable and integrated health care to those with the greatest need and the least access to service.

Vision: Empowering individuals and communities toward better health.

Values and Value Statements:

Client Centered: We deliver services and care in partnership with clients and their supports, ensuring we are responsive to individual goals and needs.

Respect and Diversity: We believe in and support the dignity and capacity of the people we serve.

Commitment to Quality: We embrace a culture of continuous improvement to deliver the highest quality care.

Health Equity: We focus on meeting the needs of those most marginalized by economic, social and health circumstances.

Compassion: We approach each client as an individual, with their own goals, needs, values and history.

Accountable: We are accountable to our clients, colleagues, organization and community through continuous monitoring and evaluation.

Target Population

Residents of Vancouver living with complex clinical and psycho social needs, who are vulnerable and underserved and who require a higher intensity of services to achieve and maintain functional stability (recognition that some clients may be homeless and unable to provide a Vancouver address but are receiving care within Vancouver).

VCH Primary Care is committed to providing culturally safe care within a framework of trauma informed practice that includes the principals of harm reduction and recovery orientation.

We collaborate and integrate with all VCH community teams (Mental Health and Substance Use, Home Health and Public Health programs), other community agencies, and acute care providers to support continuity of care for clients.

The Needs of the VCH Primary Care Clients are multi layered in their complexity and should include several of the following:

- Unattached or poorly attached despite need for primary care (i.e. no visit to provider within 18 months, no active prescriptions, no recent lab work, efforts around attachment have been unsuccessful)
- Clients attached to primary care providers but experiencing a period of functional instability that cannot be managed within a Fee for Service practice. Intent of CHC engagement would be to stabilize and support transition back to primary care provider where possible.
- Multiple social barriers such as housing instability, poverty etc. that impact on the ability to maintain a connection to care.
- Marked difficulties in accessing the fee-for-service health care system due to significant cognitive, behavioral and/or functional impairment.
- Inability to maintain lasting personal or professional relationships.
- Marked difficulties with activities of daily living without access to appropriate supports.
- Medically complex conditions presenting with chronic disease, concurrent disorders or communicable diseases (i.e. diabetes, hepatitis, HIV, mental health issues, substance misuse) that are untreated or uncontrolled.
- High emergency department use for issues that could be addressed in the primary care setting and/or frequent acute care admission/ readmission rates.
- Risk of causing harm to self or others
